# Modern management of anal incontinence

**DOI:** 10.1093/gastro/gou020

**Published:** 2014-05-07

**Authors:** Andrew P. Zbar

**Affiliations:** ^1^Professor of Surgery, Chaim Sheba Medical Center Department of Surgery and Transplantation, Ramat Gan, Israel, 52621 and ^2^Assia Private Medical Group, Ramat Hachayal, Tel Aviv, Israel

Anal incontinence (AI) reflects the final common pathway of a multitude of aetiological factors and, as an underestimated clinical problem, it represents a major social and economic burden. Its epidemiology and objective assessment of the severity of the problem are discussed in this Special Edition by Nevler from Israel. In the 1980s, the management of AI included some form of sphincter repair or scaffold, as discussed by Pescatori and Pescatori, with the construction of a colostomy as the only other surgical option. The development of a series of complicated muscle transposition procedures ensued and was supplemented by electrical stimulation, following an improvement in our understanding of the basic functioning of the voluntary myocyte. These procedures are discussed by Barišić and Krivokapić. This approach was followed by the adoption, from urological practice, of an artificial bowel sphincter that could be used to augment a perineal colostomy, in some cases of total anorectal reconstruction, and in patients with complex congenital anomalies after failed reconstruction. Neither of these more complex approaches has effectively stood the test of time, resulting in high complication rates, and the frequent need for operative revision and ultimate explantation of the device.

Given the costs (both financial and patient-related) of these more complex procedures, there are available today novel, specialized alternatives that are considered in this Special Edition. These include the use of a stoma for antegrade colonic irrigation, as discussed by Zbar (Israel), either alone or as part of a complex total anorectal reconstruction designed to augment a perineal colostomy. Also considered is the rediscovery of the use of a simple anal sling (previously used for rectal prolapse), as discussed by Devesa (Spain) and radiofrequency treatment of the anal canal as discussed by Felt-Bersma (Netherlands). The rise of primary internal anal sphincter dysfunction leading to troublesome faecal seepage was previously not amenable to therapy, with poor results from muscle plication; however, hope has arrived with the new implants—although prospective, randomized data are currently lacking concerning the ideal agent for use or the technical aspects of its optimal deployment. These issues are discussed by de la Portilla, who presents personal data from Spain.

Lately, sacral neuromodulation (SNM) has become a first-line therapy in AI management in many cases, even in the presence of small defects of the external anal sphincter, which in the past would have been repaired at the outset. The 75% overall success rate of SNM, almost regardless of the aetiology of AI (success being defined as a 50% or greater reduction in incontinence episodes), appears to be maintained over prolonged, medium-term follow-up. The technique of SNM has become fairly standardized, with the *ab initio* use of a permanent lead and decisions on permanent stimulator implantation being made after an initial period of temporary stimulation. About 60% of patients progress to a permanent implant, although there are currently no clear prognostic factors that predict a successful temporary stimulation trial. The complication rate of SNM is very low, with Zbar presenting an algorithm of its postoperative troubleshooting when functional outcomes are suboptimal. Currently, however, the simpler (and cheaper) technique of peripheral posterior tibial nerve stimulation (PNS)—performed transcutaneously or percutaneously—is reserved for SNM failures, but its true role in the treatment algorithm remains to be determined. Latterly, some comparative studies have suggested that acupuncture may be equally efficacious, and future studies will define the role of these therapies in AI cases of differing severity and aetiology.

It should not be forgotten that the first port of call in AI management involves conservative therapies and physical treatments that will substantially improve individual patient care, although the limited trial data show little improvement over placebo in terms of quality of life in collated patient cohorts. The conservative options in AI management are addressed by Dan Carter from Israel.

Finally the cost of these therapies, both in terms of economic health burden and for individualized therapy, are considered by Edden (Israel) with an emphasis on the cost-effectiveness of neurostimulatory therapies. This Special Edition clearly outlines the place of established and newer therapies in the management of patients with AI. The future will better define the indications and contra-indications, particularly for stimulatory treatments, and may elucidate the supraspinal and central mechanisms of action of neuromodulatory methods.

